# Do I belong here? The lived experience of navigating health services as a Black person living with stroke in England

**DOI:** 10.1186/s12913-025-13234-2

**Published:** 2025-08-30

**Authors:** Liz Livingstone, Tushna Vandrevala, Emily Dodd, Mary Cramp, Chunhua Chen, Andrea Drysdale, Evelyn Schiller, Melaet Habtay, Jo White

**Affiliations:** 1 Centre for Applied Health and Social Care Research, Faculty of Health, Science, Social Care and Education, Kingston University, London, UK; 2 School of Health and Social Wellbeing, College of Health, Science and Society, University of the West of England, Bristol, UK; 3 School for Business and Society, University of York, York, UK; 4 Public Contributor, Bristol, UK

**Keywords:** Health inequities, Stroke, Candidacy theory, Ethnicity, Patient-centred care, Cultural safety

## Abstract

**Background:**

Black people are twice as likely to experience stroke, more likely to have a stroke younger and have poorer outcomes than White people. Clinical factors and socio-economic status account for only half of the increased risk, suggesting systemic and structural factors are also involved. Lived experience of Black people living with stroke (BPLS) in England is under-researched. Candidacy Theory describes how people access health services, how perceptions of eligibility and suitability are socially constructed, and how this can perpetuate disadvantage. By applying Candidacy Theory to the findings of our qualitative study on the experiences of BPLS in accessing and navigating health services, we identified some key drivers of current inequalities and ways of addressing them.

**Methods:**

Our study aimed to gain greater understanding of how BPLS in England engage with and experience healthcare services. We applied Candidacy Theory to gain insight into experiences of and inequalities faced by this community and implications for service provision. 20 semi-structured interviews were conducted with adults living with stroke, identifying as African, Caribbean or of mixed heritage. Purposeful sampling included men and women with a broad range of ages and ethnicities, recruited via community and national organisation gatekeepers. Interviews were analysed using Reflexive Thematic Analysis and mapped onto the Candidacy Theory framework.

**Results:**

Findings highlighted the plethora of barriers facing BLPS in accessing services, including lack of knowledge of stroke risk or symptoms, and not being believed or listened to by healthcare professionals. While navigating services, participants encountered misalignment with their cultural and age-specific needs. Offers of services were sometimes rejected due to distrust of medical treatment, stigma associated with mental health support, lack of representation, and previous negative experiences, such as microaggression, stereotyping and racism. Participants suggested that trust-building with healthcare professionals could be impeded by historic negative experiences, further exacerbated where there is poor awareness and communication.

**Conclusions:**

BPLS face particular disadvantages and barriers in reducing their stroke risk and accessing health services post-stroke. To address these, a holistic, systemic approach is needed, to develop inclusive and “culturally safe” services, build trust and improve accessibility of services.

**Supplementary Information:**

The online version contains supplementary material available at 10.1186/s12913-025-13234-2.

## Background

Approximately 100,000 people have a stroke each year in the United Kingdom (UK) [1] making it the single largest cause of complex disability and the fourth leading cause of death [2]. Black people in the UK are twice as likely as White people to experience a stroke [3]. This risk is greater when comparing younger age groups [4], with first stroke occurring on average five years earlier [5]. Conditions which increase stroke risk such as hypertension and diabetes are more prevalent in UK Black communities [3]. Sickle cell disease (SCD), a genetic blood disorder predominantly found in those with origins in equatorial Africa, also increases stroke risk, especially in children [6]. The presentation of each stroke is unique, frequently resulting in physical impairments, but also to problems with cognition, fatigue, and changes in emotion and behaviour [7]. Experiencing a stroke can have wide-ranging consequences, impacting on relationships, families and friends [8]. Much of the existing work on stroke in Black communities has been conducted in the United States and may not reflect the situation in the UK. The majority of these studies evaluate educational interventions [9–13], with fewer studies exploring the lived experiences of those living with stroke [14, 15].

There is a lack of disaggregated UK health monitoring data for different ethnicities [16, 17]. The Sentinel Stroke National Audit Programme (SSNAP) records Black inpatients’ ethnicities as ‘African’, ‘Caribbean’ or ‘Other’ [18], and even this level of detail can be lost in analysis, where patients from ethnic minority backgrounds are combined into a single category in studies due to their relatively small numbers for comparison with Caucasian patients [5]. As an overall group, people from ethnically minoritised communities have between two and four times the risk of experiencing adverse outcomes such as severe stroke, developing pneumonia and dying in hospital [5] and have a higher level of unmet need in the years following their stroke [5, 19].

Reasons for these disparities are not clear. Traditional risk factors, clinical and socio-economic status only account for half of this increased risk [3]. Clinical studies do not always consider structural and systemic barriers involved in accessing healthcare, such as stigma, lack of awareness, discrimination and lack of trust [16]. Black people in the UK are the least likely group to be prescribed statins to lower cholesterol, and to reach healthy targets for blood pressure [5] and report lower satisfaction with their relationships with healthcare professionals (HCPs) than White patients, citing issues such as poor communication, and a lack of shared decision making and partnership [20]. In terms of engagement with post-stroke support, the Different Strokes charity notes that although approximately 13% of the UK population is of South Asian and Black descent, only 4% of those who engage with their charity are from these ethnicities [21].

Individuals from diverse ethnic backgrounds experience discrimination and disadvantage uniquely, due to the overlap of multiple social identities, with health and social inequalities which extend beyond individual characteristics, such as sex and gender. Behavioural and cultural dimensions of inequality are often overlooked, with studies failing to address the complex interplay of social, cultural, and environmental factors that shape lived experiences [22, 23]. Intersectionality considers these broad influences: social context and resources, the laws and social norms which amplify inequity, and deeper negative factors such as poverty and discrimination (the “additional burden” borne by minoritised groups) [24]. A recent rapid review conducted by the Stroke Association on stroke health inequities in the UK concluded that research may be failing to successfully represent minoritised ethnic groups and advocated for more public involvement and engagement in research [25].

While stigma, fear and distrust of services have been found to constrain help-seeking amongst Black Caribbean communities living with long-term health conditions in the UK [26] a knowledge gap exists around the particular experiences of Black people living with stroke (BPLS) in the UK, including their interactions with health services, their unmet needs and the inequalities they face.

Candidacy Theory (CT) is a useful framework to describe how people access health services, how perceptions of eligibility and suitability are socially constructed, and how this can perpetuate disadvantage amongst already marginalised communities [27]. It acknowledges how the ‘work’ of engaging with healthcare services can present further barriers to access [28]. CT characterises healthcare interactions as a series of joint negotiations between patient and provider [27]. In the *Identification* stage, an individual recognises that their symptoms need medical attention. The second stage, *Navigation*, involves awareness of the services that exist and mustering the resources to access them. *Permeability* refers to how easily a service can be accessed and considers the cultural alignment of the service and the user. *Appearance* entails asserting a claim to candidacy for the service. HCPs make decisions about the candidacy claim in a process of *Adjudication*. This may lead to the *Offer* of services, which may sometimes be *Rejected* by the patient**.** All stages are enacted under *Local Operating Conditions*, such as referral criteria, staffing, resources and relationships with HCPs. This theory has been used previously to consider the influence of factors such as gender, ethnicity and socio-economic status [29] and to explore how minoritised communities access healthcare [30–32]. Studies have also recognised that implicit power differentials between patients and healthcare providers in clinical encounters can undermine the agency of those from marginalized groups, and that patient trust is diminished by the experience of discriminatory treatment [17, 33]. The influence of past experiences and interactions on future help-seeking has been described as ‘Recursivity’ and has been posited as an integral aspect of establishing candidacy [34]. It has been argued that extending the CT model to incorporate recursivity and intersectionality enhances its explanatory potential [33, 34]. To our knowledge this is the first time that CT has been applied and extended to the experiences of BPLS in accessing healthcare in a UK setting.

## Aims

The aims of the current study were to gain greater understanding of how BPLS in the UK perceive and experience health services, and to apply CT to examine how these communities living with stroke access and navigate healthcare services. The study was part of a NIHR project (Inclusivity in Stroke Self-Management https://www.fundingawards.nihr.ac.uk/award/NIHR204975) that aimed to create resources for both BPLS and HCPs to offer better support for Black people to manage after stroke.

## Methods

### Design

The study utilised a qualitative research approach with semi-structured interviews with BPLS. Semi- structured interviews were selected as an appropriate data collection method for gaining insights into BPLS’ lived experience, feelings and opinions, offering flexibility while maintaining focus on the research question [35].

### Participant recruitment

Purposeful sampling was used to select participants with particular characteristics which could provide rich data about the experiences of BPLS reflecting a diverse range of lived experience of stroke, to include men, women, those living with SCD, a broad spectrum of ages and socio-economic backgrounds and those living with aphasia. ‘Living with stroke’ was defined both as people who had experienced a stroke and carers. This was to allow carers to participate where there were support needs (such as aphasia or low confidence) and to avoid excluding carers who were keen to share their perspective. In the majority of cases, it was those who had experienced stroke who came forward.

Inclusion criteria were adults over the age of 18 living with stroke and identifying as African, Caribbean or of mixed heritage. Recruitment was initiated via gatekeepers from community and national charitable organisations providing health support to Black communities, including those living with stroke or SCD. These included Bristol after Stroke, Caafi Health, the Caribbean & African Health Network (CAHN), the Cianna’s Smile charity, Different Strokes, Shoreditch Young Stroke Survivors and the Sickle Cell Society. Participants were also recruited via our project Patient and Public Involvement (PPI) group and professional contacts of the research team. Social media channels were used by some organisations to promote the study.

Initial contact was made via email or phone numbers provided by gatekeeper organisations and other contacts, and face-to-face at community groups.

### Ethical approval

Ethical approval for the project was granted by the University of the West of England, Bristol Research Ethics Committee (CHSS.23.10.044). All participants were given an information sheet in advance and had the opportunity to discuss the study with a member of the research team before signing a consent form, either physically in the presence of the researcher, or remotely via electronic signature. Information sheets and consent forms were replicated in an aphasia-friendly format to support the participation of those living with aphasia. A requirement of the study was that all participants had to have mental capacity to give informed consent; this was assessed by researchers when obtaining consent. Resources were available to conduct interviews in languages other than English where required, but in practice all participants were able to communicate in English. Each participant received a £15 gift voucher in recognition of their time and expertise.

### Data collection

Data was collected via semi-structured interviews. These followed a topic guide which was developed for the project and refined through two pilot interviews and input from PPI group members (please see additional file 1). Interviews explored Black people’s experiences of stroke, their views on the information and support they received, relationships and interactions with HCPs, and ideas for managing after stroke. Interviews were conducted between December 2023 and June 2024. These lasted between 40 min and two hours and were conducted face-to-face in a semi-public place, in respondents’ homes, via Microsoft Teams, or by telephone, depending on interviewee’s stated preference.

Five members of the project team were involved in data collection (CC, LL, MH, JW, AD): three university researchers and two community researchers. The interviews were conducted by a White British female postdoctoral Research Fellow with experience in qualitative research methodologies, stroke self-management, and equity of access to health services; a Chinese female postdoctoral Research Fellow with experience in qualitative research methodologies and service user engagement, and a White British female Senior Research Fellow with qualitative research expertise and experience in public involvement. Community researchers, individuals with little formal research training, can enhance the quality of research through their ability to build trust and rapport with respondents, and to uncover new insights which may not otherwise have been unearthed due to their experiential knowledge [36]. Both community researchers were members of Black communities. They received training in qualitative methods, ethics, confidentiality, reflexivity, and safeguarding. One community researcher supported three interviews by accompanying the university researcher, and one community researcher led an interview.

All interviews were recorded, transcribed and anonymised.

### Data analysis

The consolidated criteria for reporting qualitative research (COREQ) checklist was followed (see Additional File 2).

Data collection and analysis proceeded concurrently. Once data was collected, it was analysed using reflexive thematic analysis [37]. This is an appropriate approach for exploring subjective lived experience trajectories. Inductive thematic analysis was used initially to develop relevant themes from the data. Following familiarisation with the data, codes were then generated by LL and CC on participant attitudes and perspectives towards navigating the healthcare system. A subset of transcripts was read by three other team members (TV, JW, ED) to confirm initial codes and maximise the methodological and interpretive rigour of the analysis [38]. Codes were further refined through discussion with the second researcher (TV). Once a list of codes had been established, these were reassembled into themes, which related to and made sense of the connections (comparisons) between the codes. To guide the overall analysis, memos and informally written observations about participants’ experiences offering initial reflections on any potential relationships were made [39]. The themes were sense-checked in an iterative process, and in consultation with the wider research team, project Steering Group, and PPI group. Once initial themes were agreed, there was a deductive element in mapping them onto the constructs of CT. Some overlap was identified between the themes and CT sub-components, providing a useful framework to describe how people from marginalised communities access healthcare services, and how perceptions of suitability and eligibility are negotiated between patients and HCPs. The final data analysis structure was agreed with all authors.

Lincoln and Guba list Credibility, Transferability, Dependability, and Confirmability as ‘standards of trustworthiness’ for qualitative research [40]. Credibility of this research was enhanced by triangulation in the data collection and analysis process and by member checking of the results by steering group and PPI group members who were BPLS. Transferability to other settings and contexts can be determined via the detail shared about participants, verbatim quotations and transparency about our sampling and analytical methods. To ensure dependability, we have maintained an audit trail of interview transcriptions and analytical steps, while confirmability has been strengthened via diverse team involvement in data analysis, peer debriefing and member checking.

## Results

In total, 28 interviews were conducted. Eight of these were flagged as being ‘suspect’ and were removed from the analysis. The remaining 20 interviews involved 21 participants (19 Black people with a diagnosis of stroke and two family carers, one of whom was interviewed together with her husband and one of whom was the nephew of the person who had experienced stroke). See Table 1 for the socio-demographic details of the 20 Black people who had experienced stroke whose stories were shared.

**Table 1:** Demographic characteristics of participants with diagnosis of stroke

Age	30–39	2
	40–49	2
	50–59	7
	60–69	6
	70–79	2
	> 80	1
Age at first stroke	0–9	1
	30–39	3
	40–49	5
	50–59	7
	60–69	3
	> 80	1
Gender	Female	10
	Male	10
Self-Reported Ethnicity	Black British	2
	Caribbean	3
	Congolese	2
	English/Jamaican heritage	1
	Ghanaian	1
	Indian/Jamaican	1
	Jamaican British	1
	Nigerian	7
	Ugandan	1
	Zimbabwean	1
Education	Secondary School	4
	NVQs	1
	Degree	9
	Post-Graduate	3
	Not given	3
Work Status	In work	5
	Unemployed	1
	Retired	5
	Not working due to acquired disability	9
Living Arrangements	Lives alone	6
	Lives with family	10
	Lives with family, has formal carers	4

### ‘Suspect’ Interviews

The phenomenon of fraudulent participants in online qualitative research is a recently recognised issue [41]. Using social media channels to publicise studies and recruit participants may increase the risk of this occurring [42].

Eight respondents recruited through social media channels exhibited unusual behaviour during online interviews, such as declining to turn their cameras on, giving one-word answers to open questions about their stroke journey, offering similar yet atypical descriptions of their stroke symptoms or using generic language. The researchers also noted discrepancies in email addresses provided, and in one case, that a consent form from an unrelated study had been submitted. Several provided invalid telephone numbers and postal address or failed to respond to requests to provide additional contact details, as requested by the university finance departments for voucher disbursement audit purposes. Recordings of ‘suspect’ interviews were transcribed and reviewed. All were excluded from our final 20 interviews due to the generic and reticent nature of the responses.

The findings are discussed under four main themes and subthemes identified from the data: “Am I having or likely to have a stroke?” captures respondents’ perceptions about their stroke risk and symptoms, how this influenced their assertions of candidacy for healthcare services and how HCPs responded. “How do I find my way as a Black person living with stroke?” examines lived experience of engaging with services. “Why might I reject services?” embodies the personal and historical reasons that participants declined healthcare services. “Can I trust my health care provider?” summarises the factors that build and erode trust in therapeutic relationships (see Table 2).

**Table 2:** Themes and subthemes: mapping to candidacy theory

Theme	Subtheme	Candidacy Theory components
Am I having or likely to have a stroke?	• Recognising stroke symptoms • Perceptions of stroke risk • Not being listened to or believed by health care practitioners	• Identification • HCP adjudication
How do I find my way as a Black person living with stroke?	• Learning where and how to seek information • Ease (or lack of) engagement • Service misalignment with culture or age • Challenges associated with self or familial advocacy	• Navigation • Permeability • Appearance
Why might I reject services?	• Distrust of treatments and medication • Stigma associated with seeking mental health support • No one looks like me • Previous bad experiences/historical context	• Offers and Rejection
Can I trust my health care provider?	• Building a trusting relationship • (Fear of) Stereotyping • Need for Cultural Safety	• Local Operating Conditions

### Theme 1: Am I having or likely to have a stroke?

#### Recognising stroke symptoms

This subtheme relates to identifying oneself as a candidate for stroke treatment. Not recognising the signs of stroke (and therefore their candidacy) led to delays in some participants seeking help. In certain cases, they were aware of common signs of stroke because of the FAST campaign – that is, Facial droop, Arm weakness and Speech difficulties [43]. However, because their stroke presented differently to these signs they did not immediately seek help:

*Sometimes they’re like, oh, your face falls or like, there’s slurred speech, and there’s this, that and the other…. or loss of mobility and stuff, I didn’t really have that* (P17, Black British/Caribbean, 37 years, female)

Further, participants living with SCD described signs of stroke as being hard to differentiate from other symptoms related to their condition.

In many cases, a friend, family member or social worker was instrumental in persuading the participant to go to hospital. Conversely, other participants were convinced by a loved one that they did *not* need to seek help. One participant described experiencing a second stroke, which presented completely differently from her first. It was her belief that it was not possible to have a second stroke. Her sister agreed:

*I woke up and noticed that my left arm was very heavy and numb and then I called my sister and said this is how I am feeling. And she’s like, you probably slept on your arm. So don’t worry* (P4, Ghanaian, 62 years, female)

#### Perceptions of stroke risk

Failure to identify candidacy was also linked to perceived risk of having a stroke. Many participants described low awareness of the increased stroke prevalence and younger onset in Black communities, or of the stroke risk presented by co-morbidities such as hypertension. This included a respondent who had worked for decades as a professional nurse and was unaware of her heightened stroke risk. Consequently, risk factors were often not being managed. Many participants had been unaware that they had one or more of these underlying conditions until after experiencing stroke.

*Later, I would ask him – what’s the cause of this stroke? It was later they told me that it was due to high* cholesterol *and irregular heartbeats* (P2, Nigerian, 55 years, female)

In some cases, participants revealed that they had not taken up offers of screening appointments, which could have helped identify conditions which increase the risk of stroke, such a high cholesterol, high blood pressure, diabetes or irregular heartbeats. Others had been aware that they had co-morbidities but did not perceive these as presenting a serious health issue, because they felt well and were unaware that taking prescribed medications would help reduce stroke risk. One participant described taking medication for hypertension sporadically, or not at all, for this reason. His doctors attributed his stroke to this.

*I never think I will have a stroke because I’m a thin guy...I didn’t have high cholesterol. When they found out I was on high pressure. I was taking tablets for blood pressure. But to be honest, that tablet blood pressure, I was taking it not in a proper way. I’m going to take that medicine like Monday, Tuesday, I can miss maybe Wednesday...I got negligent. I don’t know. Then when I said that, the doctor say, it’s that what’s caused your stroke* (P6, Congolese, 65 years, male,formerly living with undiagnosed hypertension)

Participants living with SCD described a lack of information and knowledge of their heightened stroke risk.

*I know that if I knew what I know now about stroke, I wouldn’t have got a stroke* (Participant P1, Ugandan, 51 years, female)

*I didn’t know about it until it happened to me. There wasn’t a lot of information really about it* (P10, Nigerian, 33 years, female)

Some participants described health beliefs such as ‘stroke happens to old people’, or that young, slim and apparently fit individuals were not at risk.

*Before I was thinking, it happens only on older people but when I went to the hospital, even the young are… There are some young guy, 25 years old* (P6, Congolese, 65 years, male)

#### Not being listened to or believed by health care practitioners:

Having identified their candidacy for treatment, participants described their attempts to engage with healthcare services. Some described the ease of accessing services, while others reported being initially misdiagnosed and, in some cases, turned away by services. One participant recalled presenting at the Accident and Emergency (A&E) department feeling very unwell, but because she was vomiting, she was diagnosed as having a ‘stomach bug’, given fluids and then discharged. Another recalled being initially misdiagnosed over the phone as having vertigo.

*We phoned the doctor, and, they said, ‘oh, it’s probably vertigo’, and I was like, ‘I’ve never had vertigo in my life, what is vertigo?’ And then, after like two days, I had to go to hospital *(P12, Caribbean, 41 years, female)

In these cases, HCP rejection of the participant’s candidacy as a person experiencing stroke delayed time-critical treatment by more than 24 h. Some respondents related such experiences to their being less likely to be listened to, taken seriously and believed because they were Black. One participant commented on how there was little point in raising awareness of stroke via campaigns such as FAST if patients were being turned away from hospital. She reflected on how negative experiences create or compound distrust and may result in people failing to engage with services in the future:

*If people do come in because they know something is wrong but you’re not being believed, then it just kind of defeats the purpose...They’re going to A&E and they’re not feeling well and they know something is wrong, but the A&E doctor doesn’t believe them…. And then later on that’s obviously going to affect how they access services moving forward?* (P10, Nigerian, 33 years, female)

In a further, distinctive example of rejection of candidacy, one participant was wrongly identified as being an irregular migrant, with no right to free hospital treatment. He described receiving acute care then being presented with a huge bill and denied further treatment; an extremely stressful experience. By the time this dispute had been settled it was too late for him to benefit from further, post-stroke rehabilitation.

*You know, before they discharge you, they will take you to a home where you do therapy to know how to use your hands and body and everything again, to know how to speak and all that. They denied me, they said, because I don’t have paper. That is why I can’t use my hand. I can’t use it because nobody has cared for me* (P9, Nigerian, 58 years, male)

This participant’s already difficult experiences were compounded further by the overtly racist behaviour of a carer provided by the council, who told him *‘pack your loot and go back to Africa’.*

The theme above describes how the first step in accessing healthcare services for BPLS is self-recognition of a legitimate claim to candidacy for medical attention for stroke, including preventative measures to manage stroke risk. HCPs (and in the case cited above, wider service) recognition of the candidacy claim is also instrumental. When claims are not believed or taken seriously by HCPs this presents an insurmountable barrier to accessing services. The examples outlined where HCPs ‘adjudicated’ against claims had significant health outcomes in terms of delayed access to emergency care and post-stroke rehabilitation.

### Theme 2: How do I find my way as a Black person living with stroke?

In describing their experiences of navigating services post-stroke, respondents highlighted how accessibility was influenced by knowledge about services, having the resources to use them, and ease of engagement. There was work and effort involved in making candidacy claims, often requiring support from family members. A lack of alignment with the specific needs of BPLS sometimes presented barriers to engagement. On occasion, the services offered were rejected, for reasons which included historical mistrust, cultural and health beliefs or misalignment with needs.

#### Learning where and how to seek information

With little or no prior knowledge about stroke and services, participants emphasised the need for trusted and relatable information. Some reported having been given excellent guidance by HCPs after their stroke. However, many described confusion and frustration when looking for information and navigating services, with a lack of signposting and guidance about what was available to them, especially early in their stroke journey. In some cases, participants suggested that they had been given the opportunity by their HCP to ask questions, but needed more guidance on where to begin, what might be helpful for them to know, and the relevant resources available.

*The whole thing is, it’s very confusing. You go onto the internet and then you get some information. But everybody is saying something different* (P4, Ghanaian, 62 years, female)

*I think it’s because you don’t have a starting point of what to, what not to do and what to do, so, you, kind of just like, I don’t really know where to start* (P12, Caribbean, 41 years, female)

Participants recalled often not knowing what they needed from support services shortly after a diagnosis of stroke. Here, a participant describes an unhelpful interaction with social services shortly after her young husband’s stroke.

*Adult social care just came to see us when he first came home…. Like, “what do you need”? I said I don’t know…I have no idea. I’ve not done this before.” But you need to tell me what you need”. I said, I don’t know what that means. We called you for support…. I don’t know what I need. Like, what’s available? It was a very frustrating conversation. I just thought so why did you come? …. I’ve told you a situation. I don’t know what resources. I have no idea. I haven’t got a clue as to what way you can help me* (P20, Nigerian, 44, female; wife of P19).

She contrasted this with an extremely positive experience after contacting a charity specialising in Stroke.

*I didn’t know what I was calling for… I just said my husband’s had a stroke and I don’t know what to do.…. she was amazing…so kind and understanding. she was very calm in her approach... And the way she spoke, she understood. Ask the right questions... that made all the difference. *(P20, Nigerian, 44, female; wife of P19)

In another example, a participant recalled feeling ‘shut down’ by a physiotherapist, making her reluctant to ask him questions. As a result, she felt strongly that BPLS need to feel supported to ask questions and become informed about their care.

*Anything I ask, he makes it look like you are trouble’…’ the answer he will give you makes you not want to ask another one… If it does happen [a stroke], you make sure you ask questions and know more about it* (Participant P4, Ghanaian, 62 years, female)

The importance of empowering BPLS was echoed by another participant who also highlighted the value of culturally appropriate information:

*The confidence to ask I think is what we need to give people.. I don’t think I’ve seen anything, with regards to stroke, within the Black community or kind of, any person of colour... I’ve seen kind of, stroke information, just generally, but it’s always kind of, geared to kind of, in the nicest possible way, White culture* (P12, Caribbean, 41 years, female)

#### Ease (or lack of) engagement

Several participants also reflected on the low permeability (or ease of engagement) of preventative services. The timing and location of GP appointments and screening services presented barriers to monitoring their health (and consequently managing their stroke risk), especially if they were working long hours or engaging in shiftwork.

*A lot of Black people as well they don’t want to go see their GP or it’s really hard to get a GP’s appointment* (P10, Nigerian, 33years, female).

*We don’t have time to check our BP [blood pressure]. To check whether we have diabetes, whether you have sickle cell* (P2, Nigerian, 55 years, female)

Several participants described dissatisfaction with GP services, for their lack of continuity of care, and short appointment duration, allowing insufficient time to discuss issues properly, which acted as disincentive to seeking regular checkups.

*‘Cos if you go in there, it’s about five minutes and it’s like a, a, conveyor belt, you know?* (Participant P18, Caribbean, 77 years, male)

*I should do (regular GP check-ups), but I don’t really, probably cos it’s a really bad GP service... I don’t really have, like, a set doctor, so, like, every time I go to the doctor’s surgery, I have to explain everything all over again* (P12, Caribbean, 41 years, female)

#### Service misalignment with culture or age:

Permeability of services is also influenced by the extent to which they align with the candidate’s cultural practices and values; misalignment creates further barriers to access. In a clear example of misalignment one participant described having her personal care needs assessed for Personal Independence Payments (PIP). The assessor could not understand why she was unable to shower independently.

*If the stroke affects like a limb, Afro hair is different than European hair…If your limbs are affected, you, you can’t braid, and that affects kind of, like how you wash your hair and stuff. So, you always have to have someone else wash your hair for you…. I remember when I was speaking to somebody at PIP and they said like, can you have a shower yourself? And, I was like, if I have help getting in, I can have a shower myself, but I can’t wash my hair. And, they said, “Well, why not?” And, I was like, well, because I have Black hair. And, it’s something that didn’t kind of click* (P12, Caribbean, 41 years, female)

Other participants raised the issue of dietary advice generally being based on a Western diet, with little regard for non-Western traditional foods. Paying attention to small details could enhance sense of inclusion.

*They need to cater for everyone… They [the hospital] did have some halal meals and some ethnic meals, yes, but a little bit. But that does go a long way because it shows that you’re being included... I don’t think that society knows that there’s some invisible barriers for people who are disabled or who are of colour. You don’t see those barriers until it’s happening to you, then you realise there’s a barrier. But they are not visible...What they’re doing, they’re damaging people because what they’re doing is not applicable... You could change it by asking people to speak about their experiences and the barriers they have experienced because of the Eurocentric approach* (P3, Zimbabwean, 60 years, female)

Almost half of our participants experienced their first stroke under the age of 50, reflecting Black people’s higher risk of stroke at an earlier age. Younger participants described issues with permeability of services because of their age as well as their ethnicity. One participant in his 40 s described feeling that he did not belong on the stroke unit, because other patients were substantially older. His wife felt, similarly, that the service was directed towards the needs of older, less active (and possibly less demanding) patients.

*The fact I was the youngest person that I think they didn’t, they probably didn’t really know what to do with me to be honest* (P19, Nigerian, 47 years, male)

*I got the impression they were used to people just older people having strokes and no questions being asked… I mean, especially with strokes happening to younger people, younger people needs to be treated different. The care is still designed for somebody that’s 60 years plus, but if it’s happening to 40 years and under, the rehabilitation is very different. The mindset of that person is very different* (P20, wife of P19)

When this participant was being discharged from hospital, his healthcare team tried to find him a place in an inpatient rehabilitation unit. This was unsuccessful, because he was categorized as too high-functioning, with goals that did not fit the referral criteria of the unit. Service misalignment with his needs as a younger, more active man resulted in HCPs rejecting his candidacy claim.

*You’re not suitable candidate because you’re not... you weren’t sick enough…I did wonder. How maimed do I have to be??* (P19, Nigerian, 47 years, male)

#### Challenges associated with self or familial advocacy

It was clear from our interviews that for BPLS, engaging with and learning how to navigate services involved substantial time and energy, compounded by a lack of trusted information and open dialogue. Several participants described the effort of acting for themselves, chasing test results and driving their own care, with a sense that no-one was acting as a consistent advocate for them. This impacted them in various ways, including engendering further distrust with services.

*Myself, I have to follow, to push. I have to dig for it. All the time…every single thing* (P6, Black African 65 years, male)

*I just don’t think that my surgery was well versed enough for stroke victims, to be able to offer me the service, that I probably needed. I’m just angry at the situation... because you can’t really, like have a starting point on what you need to do improve you’re always at that kind of, no man’s land, where I’m just like, what do I do next?... It’s kind of, um, powerless, you don’t know what to do?* (P12, Caribbean, 41 years, female)

Participants described the vital support role played by friends and family. However, the effort of navigating services could be burdensome, with family members describing how they needed to be more involved in decision-making, as well as requiring support themselves:

*I think just a first point of call it would have been nice just to have the one person I could go to …. instead of repeating the conversation again and again …Nobody sat me down and said, ‘look, he’s coming home. What do you think he’s gonna need? Can I come to your house and see you. Do you work? How are you working? How are you getting on? Are you going to be able to go to work?’* *The family can help you help them, you know?* (P20 wife of P19)

Several participants described changes to family life; parents retiring or relatives coming to live with them to support them. One participant had become the main carer for a male relative whose adult children and close family were overseas.

*So it’s just the wife and I, you know, left with the responsibility of looking after him, particularly the first few weeks. So I arranged my life’ … It was quite traumatic you know, not just for him but for us as well as a family* (P11, nephew of Nigerian 73 years, male)

Impacts can thus ripple out to extended family. This participant also commented that some first-generation migrants may lack family networks in the UK to provide support after stroke.

To navigate healthcare systems, BPLS need trusted information about the services that are available to them. Certain services were less permeable to some of our respondents, either because they were logistically harder to engage with, or because they were misaligned to their specific needs. Families often provided vital support in the ‘work’ of navigating services but this came at some personal cost and lack of communication and joint decision-making with HCPs was highlighted as exacerbating this.

### Theme 3: Why might I reject health services?

Although some candidacy claims were rejected by HCPs (as discussed in Theme 1), in other cases participants themselves refused services that were offered. This was due to distrust of treatments and medication, cultural stigma associated with seeking mental health support, lack of representation within services and previous experiences of racism, discrimination and stereotyping which had created distrust.

#### Distrust of treatments and medication

Screening appointments and medication were sometimes refused because they were not considered relevant or important, particularly where participants perceived themselves to be young and fit and not at risk of stroke. In other cases, participants were distrustful of treatment.

*Then he started treating me with the diabetes treatment, but up ‘til last year, I told him I will not take insulin because I don’t know the consequence of insulin (P9, Nigerian, 58 years, male)*

#### Stigma associated with seeking mental health support in my culture

Some cultural attitudes and beliefs contributed to refusal of services, particularly those relating emotional and psychological support, where there was a cultural stigma relating to mental health and seeking support in this sphere:

*When people were saying, okay, we’re going to refer you … what do they call it? For the mental thing,? ….. So I was like, oh, they feel I’m mad?* (P6, Black African, 65 years, male)

*You’ve grown up in a Nigerian household like I did, mental illness I think is something they don’t… Not that they don’t really understand, but it’s a lot of like everything with them is like, oh just pray, pray, pray, pray, it will all be all right.* (P10, Nigerian, 33years, female)

#### No-one looks like me:

Participants described rejecting offers to attend peer support groups where ‘no-one looked like them’. This was often related to both age and ethnicity.

*There was supposed to be a younger stroke group, but it seems like, they’re still a lot older than me... It’s a different experience for me, especially having young children as well, than it might be for someone who’s older, has grown children, has different responsibilities….Might be retired, so, they don’t need to think about going back to work…It’s a different life stage and a different experience of what, how this stuff is impacting... I went to the, to the second group, ‘cos that had more Black and Brown people in there... it would be nice to find something that is within the Black culture, and, also within kind of, my age group* (P12, Caribbean, 41 years, female)

#### Previous bad experiences

Some of our participants revealed how in their community, previous negative experiences, such as racism, discrimination and stereotyping had created a general sense of mistrust which could lead to refusal of services.

*I think most of the time they try and take care of their own. I feel like sometimes, people can be untrusting of services, I think, due to racism and bad experiences that they’ve had, within health services* (P17, Black British Caribbean, 37 years, female)

*As a Black person, you could just tell somebody who is warm towards you or somebody who isn’t* (P3, Zimbabwean, 60 years, female)

Respondents rejected services because of cultural stigma, distrust or the feeling that they did not belong. The bar for rejection was potentially set lower because of previous negative experiences. Some suggested that acceptance of services could potentially be increased by working in collaboration with established community organisations, who were considered trusted sources of information.

### Theme 4: Can I trust my healthcare provider?

#### Building a trusting relationship

The degree of trust and confidence that participants had in their relationships with HCPs influenced access to and uptake of services. Many participants described how the quality of interactions influenced their experience of care. Developing trust could be made harder by previous negative experiences, as described in Theme 3. Continuity of care helped trust to develop over time. One participant described how her long-standing GP gave her an (unheard of) 30-min appointment, to allow her to fully discuss her issues post-stroke, while others described the supportive and collaborative approach of their stroke rehabilitation teams, prioritising the needs of the patient.

*She is just somebody that I could speak to and explore my health issues and how my health issues are affecting the rest of my life and what I and what I could do about it* (P1, Ugandan, 51 years, female)

*They assisted me to get back to myself’* (P2, Nigerian, 55 years, female).

*They made me feel like they’re here for you’* (P4, Ghanaian, 62 years, female)

*A lot of it is just about putting the patient at the centre of the care and working with the patient* (P10, Nigerian, 33 years, female)

For several participants, however, poor communication and inconsistent messages had negatively impacted their relationships with HCPs.

*Before, I used to think the healthcare professionals were helping. But up to some point, I stopped trusting them, believing them, because they don’t seem to be consistent* (P4, Ghanaian, 62 years, female)

Another participant described asking his hospital doctors how long he would take to recover and being told ‘they didn’t know’. He was shocked to subsequently be told, with a couple of days’ notice, that he was being discharged from hospital. It was only then he realised that he would be discharged despite still having some disability.

*“You’re going to go home soon. You can’t stay here”. I said, I’m not all right, how can I go home like this?… They said…”you must understand is there’s so many people who got situation like you. If you stay here, no space. So what you going to continue your sickness, your home”. I said ‘oh my God’ (P8, Congolese, 62 years, male)*

Some participants expressed suspicion about healthcare professionals’ underlying motives. They questioned whether or not they were getting the ‘the full story’ or being offered all treatment options and wondered if this was due to their ethnicity. One participant described his reaction to hearing about the various services offered to others at his peer support group. His suspicions about being treated inequitably further exacerbated his distrust in HCPs.

*When they ask me a question, I feel something bad behind…If you ask me a question,* *it’s maybe something they’re not telling me. That’s always I’m asking myself. I* *don’t know if they say the truth… I think because maybe I’m black, that’s why. …* *When I go to the group, I ask them. They say, oh, for that problem they sent me to do* *this, they sent me to do that, I had this medication, blah, blah, blah…but me, formyself it’s not the same (P6, Black African, 65 years, male).*

#### (Fear of) stereotyping

Stereotyping has the potential to influence HCP adjudications. Some participants had directly experienced racism and being stereotyped in healthcare interactions and described having to be guarded about their behaviour for fear of being labelled. Eroding trust in this way potentially impacted on future engagement and acceptance of offers of services, contributing to a sense of distrust.

*Because they think ‘you’re black’, they put you in a box or they give you medication* (P3, Zimbabwean, 60 years, female)

*You just know there’s an element of racism in there and I can only say this as my experience as a black woman, the way I get treated compared to other people is very, very different...knowing that I can’t get angry if I don’t receive good care because if I do get angry, I’m going to be labelled as something – an aggressive or Angry Black Woman. And it’s just so unfair that we have to navigate that daily* (P10, Nigerian, 33 years, female)

#### Need for cultural safety

Creating a safe space in therapeutic encounters was seen as important, particularly to counter distrust resulting from historical experiences. Participants discussed the need for HCPs to recognise and be sensitive to these vulnerabilities:

*There’s this historical backdrop to these things, and that’s not going to just go away because we want it to go away. It’s a process that we have to work through… the more people interact and reassure, because the Black community in particular, they need reassuring giving the historical backdrop of everything that has happened. So professionals need to be aware of that* (P11, Nephew of Nigerian, 73 years, male)

Several participants talked about the need for HCPs to reflect on potentially ethnocentric attitudes in their practice. For many of our participants, trusted relationships could be harder to develop because of historic experiences of racism and stereotyping. This could be exacerbated by healthcare professionals’ poor communication and lack of cultural competency.

*Professionals need to be taught they need to look at themselves. You cannot enforce your values from your own background onto other people who are not the same* (P3, Zimbabwean, 60 years, female)

*One thing they should understand is we all come from different backgrounds and different cultures and everything. And we should try and adapt…And then we have to learn from each other.* (P4, Ghanaian, 62 years, female)

It was also suggested that having more visibly diverse healthcare teams would be helpful in creating trust:

*Representation does matter. I think it, when people are seeing more people of colour, within those settings, it makes them feel a lot more comfortable and actually, more comfortable, confident to open up* (P17, Black British Caribbean, 37 years, female)

## Discussion

The aims of the current study were to gain greater understanding of how BPLS in the UK perceive and experience health services and to apply Candidacy Theory to encompass how they navigate healthcare services. To the best of our knowledge this is the first study to explore the lived experience of BPLS in the UK and identify the factors and unmet needs related to risk awareness, health service provision and navigation which are contributing to the current health inequalities they face in relation to stroke.

The study makes a novel contribution by reflecting the lived experience and unmet needs of BPLS in the UK, and how personal, historic and systemic factors influence their candidacy as users of healthcare services. Our work has emphasised those elements of Candidacy Theory that are particularly pertinent to BPLS namely (1) issues with identification as a candidate for stroke and prevention services, (2) reduced permeability of services due to cultural misalignment with age and ethnicity, and (3) the influence of previous bad experiences, stigma and historic mistrust. Candidacy theory supports the idea that inequities of access arise from healthcare services which are constructed around an implicit ‘ideal user’, with characteristics that meet with referral criteria, needs that match with how a service is intended to be used, and preferences which align with the way the service is delivered [27]. The current study has highlighted how structural and systemic factors influence how BPLS navigate healthcare systems. Black people face a double disadvantage of heightened risk of stroke yet limited awareness of this risk. Issues around identification as a candidate for post-stroke and preventative services impacted help-seeking and subsequent outcomes. The belief that ‘stroke happens to old people’ was widespread. This finding is noteworthy, given stroke occurs around five years earlier in Black communities than White communities ​[4, 5] ​and those living with SCD have a greatly elevated risk of stroke, even in childhood ​[6].

Our work draws attention to the reduced permeability of stroke services due to misalignment with age, culture and ethnicity​. For some BPLS, services were considered less permeable due to them not feeling represented both as a Black person and a younger person—this intersectionality has not been considered in previous studies on stroke in the UK. Increased permeability was associated with continuity of care and the efforts made by HCPs to give time to BPLS to discuss their needs in detail.

Like previous studies [17, 33, 34]​, we found recursivity to play a key role in candidacy, with prior poor experience, including racism and stereotyping, and historic mistrust influencing both the rejection of services and the nature of any interactions with HCPs. An additional element identified was the sense of unease reported in some encounters and the requirement to self-regulate behaviour due to a fear of being labelled. Frustration about HCP assumptions regarding cultural ‘norms’ highlighted the need for more inclusive service design and training and greater self-reflection amongst professionals. Previous studies have highlighted the importance of a ‘safe space’ for patients to address current inequalities and our findings describe the ways in which current services often fail to provide this for Black people living with stroke [44].

### Implications for service design and clinical practice

**Fig. 1:**
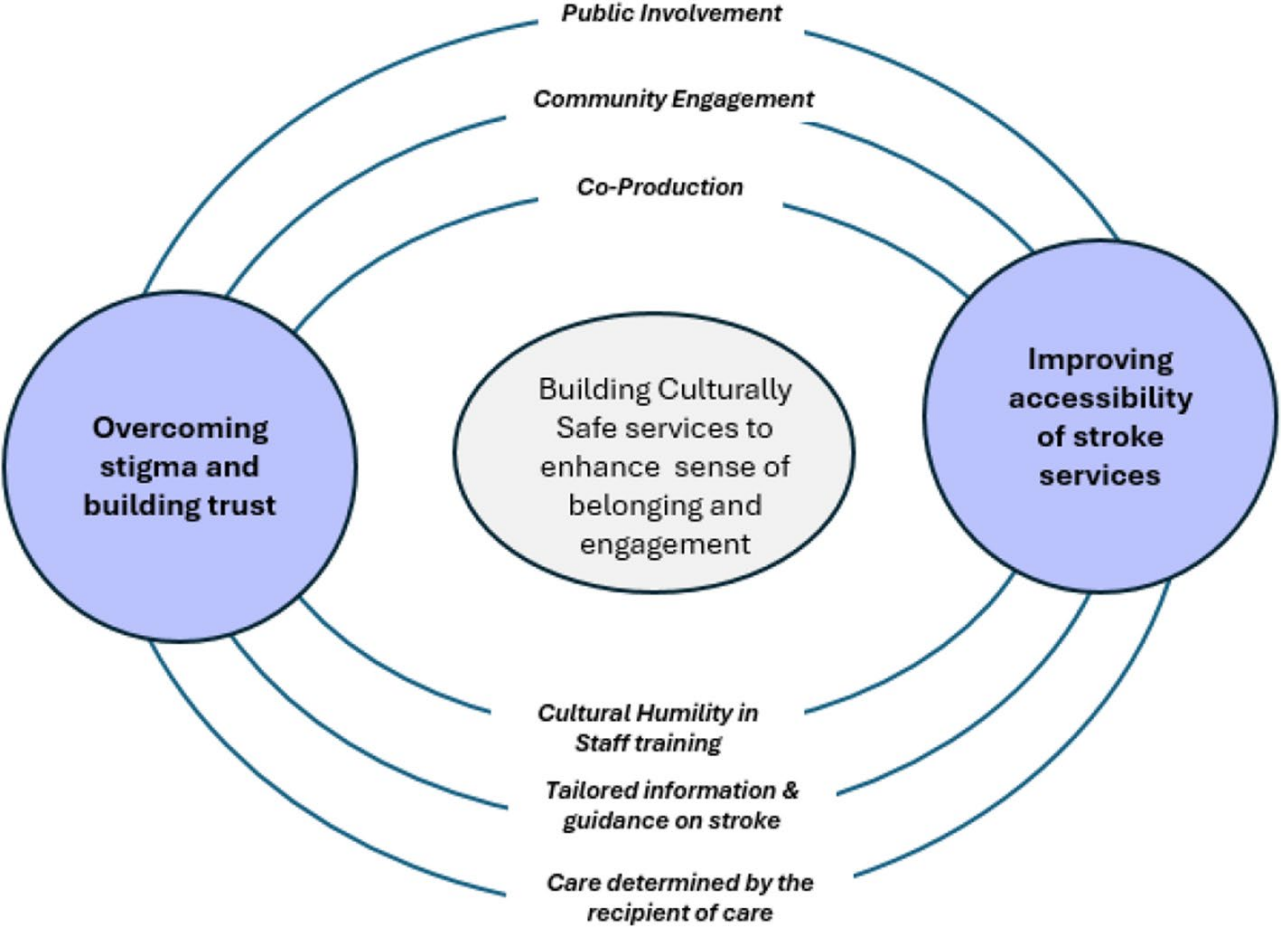
Developing Inclusive and “culturally safe” clinical practice and services

Our findings have implications for clinical practice, service design and commissioning of stroke services for Black communities (see Fig. 1). A recent synthesis of stroke experiences of those of African descent living in high-income countries highlighted the need for individualised, intersectional and ‘culturally humble’ approaches, which move beyond notions of cultural competency, in order to address unmet needs and wider problems of trust in HCPs [14]. The Cultural Safety approach extends this further, arguing that due to institutional racism, there is failure to recognise cultural difference as normal, or to provide for it. To address this, it advocates using reverse innovation—learning from those who have historically wielded less power—such that care is determined by the recipient, with service users deciding if their care is culturally safe for them [45].

Our study draws attention to the need to use concepts of cultural safety in service design and professional training to embed critical reflection amongst service commissioners, managers and HCPs of the impact of their own culture, beliefs, values, biases, assumptions of power, and the role of historical white privilege on the clinical encounter. To address systemic challenges, services need to be structurally reflexive, conducting critical conversations about institutional biases and racism to identify the blind spots generated by historical power imbalances. The use of public engagement and co-production, and active listening to diverse patient experiences are essential to address issues of racism, lack of cultural humility and empathy. The National Stroke Service Model mandates the involvement of stroke survivors in discussions and decision, to ensure that services are co-designed and meet the needs of users [46] and that the voices of minoritised ethnic groups are represented [25].

### Overcoming stigma and building trust

Distrust and mistrust describe distinct phenomena [47]. Distrust derives from informed opinion and personal experience, whereas mistrust pertains to more generalised sense of unease or suspicion. ​Our findings suggest that refusal of services can be due both mistrust and distrust—to historic experiences and cumulative microaggressions, but also to cultural beliefs and dislike or mistrust of medication and medicalised care. There may be suspicion of HCP’s motives, or stigma around mental health support. Stigma, fear and mistrust of services have been found to constrain help-seeking in Afro- Caribbean and other ethnic minority communities in the UK [26] and historic injustice is known to undermine engagement by engendering broader mistrust of institutions [48, 49]. HCPs may understand the need for culturally competent (rather than culturally safe) practice, while lacking confidence in their ability to provide it [20]. Tackling this, with more training and resources, is seen as vital in addressing health inequities. HCPs have a duty to reflect on their role as gatekeepers and adjudicators of access to services [30]. They have a responsibility to acknowledge the historical context, mistrust and additional work of making a claim where services are poorly culturally aligned [26]. By making efforts to develop trusting therapeutic relationships, HCPs have the potential to reshape care experiences, recursivity and perceptions of future candidacy [34]. ‘Recursivity’ describes the influence of past experiences and interactions on future help-seeking. Our findings highlight how beliefs such ‘I won’t be believed or listened to’, or ‘they don’t know what to do with me’ can impact on future assertion of candidacy amongst BPLS. Our findings also highlight that legitimacy of candidacy can be challenged when no-one in the peer group or information guidance ‘looks like me”.

### Improving accessibility of stroke services

This study highlights multiple examples of misalignment between service and user, namely how culturally aligned a service is with the user’s needs and values relating to ethnicity and age. For example, Eurocentric bias is evidenced in dietary advice and the needs of younger BPLS being poorly served, with some services having a ‘ceiling’ of goal achievement which is inappropriately low. It is recognised that services which appear to be broadly permeable or accessible may be less so to certain groups, unless their specific needs are considered [28, 31, 34] and patients who are seen as undeserving in some ways are more likely to be excluded [28, 31]. Participant trajectories highlight how stereotyping impacted on their care—being ‘put in a box’ because of being Black, being prescribed unwanted medication, or having to modulate their behaviour for fear of being judged to be an ‘angry Black woman’.

Furthermore, our findings highlight the lack of perceived personal risk of stroke and the difficulties faced by younger members of Black communities in engaging with preventative services. Some participants were aware of the FAST campaign but did not associate it with their symptoms or relate it to their age group. Health education campaigns, such as FAST raise public awareness but can also create unintentional barriers, where the advice provided is not considered personally relevant [1, 50]. There is a need for specifically targeted campaigns to raise stroke awareness amongst younger subgroups and in culturally and linguistically diverse communities and current campaigns and education need to be comprehensively reappraised to meet their specific needs [4, 51]. These need to address culture-based explanations and perceptions of risk, such as stroke being caused by ‘too much thinking’, the propensity to habitually downgrade warning signs of illness, only seeking help when it has become medical emergency, as well as fear of dismissal or judgement by HCPs. Community engagement and partnering with established community organisations may offer a route to more effective communications in this sphere.

### Study limitations

These findings begin to illustrate the complex factors that influence navigating stroke services for Black communities in England. By their nature, these findings are specific to a particular minoritised group in contact with community organisations, and moving forward, future research needs to test the transferability of our results. Our data was collected from two ethnically diverse English cities in the south of England. Efforts were made to include participants with a broad range of ages, ethnicities and experiences, including living with aphasia and SCD. Most participants lived in urban areas and had high levels of educational attainment, and their experiences may not be representative of those living in less ethnically diverse or rural areas and/or those with lower socioeconomic status. Despite working with several community organisations, we experienced difficulties with recruiting participants from more marginalised communities, which may reflect broader distrust of institutions. Our community researchers, PPI group and representatives from community/gatekeeper organisations from Black communities were consulted in the development of topic guides, initial findings and themes to sense check and validate our findings. Given the issues with ‘suspect’ interviews, we would not use social media channels for recruitment in the future but would continue to work directly with community partners.

## Conclusions

This qualitative study used Candidacy Theory to provide new insights into how Black people living with stroke perceive, experience and navigate health services in the UK. Although Black people are more likely to experience stroke than the White population, little literature exists from the UK in this area. Our findings suggest how personal, historic and systemic factors influence Black people’s candidacy as users of healthcare services post-stroke. The participants in this study reported mixed experiences with interactions with healthcare professionals; from very positive experiences to stereotyping microaggressions to experiences of explicit racism. Those negative experiences perpetuate disadvantage and inequalities in access to stroke care. To improve the experiences of BPLS in interacting with healthcare systems, the structural and systemic factors influencing those experiences need to be addressed to develop culturally safe and competent practices, through addressing cultural misalignment of services and the historical mistrust felt towards institutions. Overcoming stigma, building trust and improving accessibility as described in Fig. 1 are mutually reinforcing and linked to recursivity—the influence of past experiences and interactions on future help-seeking. Making changes to future help-seeking experiences is crucial to addressing inequalities facing Black people living with stroke over the longer term.

## Supplementary Information

Additional file 1. Topic Guide. Description: Topic guide used in semi—structured interviews. 

Additional file 2. Title: COREQ checklist. Description: Consolidated criteria for reporting qualitative studies (COREQ): 32-item checklist

## Data Availability

The datasets used and/or analysed during the current study available from the corresponding author on reasonable request.

## Abbreviations

A&E: Accident and Emergency
BPLS: Black people living with stroke
CAHN: Caribbean & African Health Network
CT: Candidacy theory
HCP: Healthcare professional
ISSMAS: Inclusivity in Stroke Self-Management
NIHR: National Institute for Health and Care Research
PIP: Personal independence payment
PPI: Patient and public involvement
SCD: Sickle Cell Disease
SSNAP: Sentinel Stroke National Audit Programme
UK: United Kingdom
